# Evaluation of brachial plexus stiffness in different arm and head positions by sonoelastography

**DOI:** 10.1097/MD.0000000000035559

**Published:** 2023-10-13

**Authors:** Muhammet Ahmet Karakaya, Kamil Darcin, Ilker Ince, Yilmaz Yenigun, Kamber Kasali, Aysenur Dostbil

**Affiliations:** a Department of Anesthesiology and Reanimation, Koc University Hospital, Istanbul, Turkey; b Department of Anesthesiology and Perioperative Medicine, Penn State University, Milton S Hershey Medical Center, Pennsylvania, USA; c Department of Biostatistics, Faculty of Medicine, Ataturk University, Erzurum, Turkey; d Department of Anesthesiology and Reanimation, Ataturk University Hospital, Erzurum, Turkey.

**Keywords:** brachial plexus, injuries, sonoelastography

## Abstract

Intraoperative positioning-related nerve injuries, particularly those affecting the brachial plexus, are concerning complications believed to arise from stretching and/or compression of peripheral nerves. Although sonoelastography, a new ultrasound technology, is emerging as a valuable tool in the musculoskeletal system, its utility in evaluating peripheral nerves remains unclear. This study aimed to utilize sonoelastography to assess the brachial plexus during surgery, specifically investigating changes in its stiffness values in relation to different head and arm positions. In this prospective cohort study, bilateral brachial plexuses of 8 volunteers in 3 different positions were enrolled. Using a high-frequency linear probe, the stiffness of the brachial plexus was quantitatively measured in kilopascals (kPa) under 3 different positions: neutral, head rotated, and head rotated with arm hyperabducted. Intra-class agreement was evaluated. The stiffness of the brachial plexus was 7.39 kPa in the neutral position (NP), 10.28 kPa with head rotation, and 17.24 kPa when the head was turned, and the ipsilateral arm was hyperabducted. Significant increases were observed in stiffness values when the head was turned, whether ipsilaterally or contralaterally, and during hyperabduction of the arm while the head was turned (for all *P* < .001). Strong intra-class correlations were found for the measurements of stiffness values (ICC = 0.988–0.989; *P* < .001; Cronbach Alpha = 0.987–0.989). Sonoelastography revealed significant increases in the stiffness of the brachial plexus with various head rotations and arm positions compared to the neutral state. These findings suggest that sonoelastography could potentially serve as a valuable tool for assessing the risk of brachial plexus injury during surgery and for guiding optimal patient positioning. Further research with larger sample sizes is needed to establish definitive clinical applications.

## 1. Introduction

Intraoperative positioning-related nerve injuries are postoperative complications that are believed to arise from the stretching and/or compression of peripheral nerves.^[1]^ These injuries are commonly encountered in routine clinical practice and can occur in any peripheral nerve. However, one of the most frequently injured nerves during surgery is the brachial plexus.^[1]^

The etiology is not clearly defined, but cadaver studies have shown that the brachial plexus is compressed at the level of the scalene muscles, between the ribs and the clavicle, or stretched along the head of the humerus in certain positions. This has been found to increase when the head is turned to the opposite side. Additionally, it is known that hyperabduction of the arm may cause the head of the humerus to compress the brachial plexus.^[1]^ Various imaging methods can be used to assess brachial plexus damage, one of which is sonoelastography.

Sonoelastography is a technique that emerged as a new ultrasound technology, which qualitatively and quantitatively measures the elasticity of tissues.^[2,3]^ Numerous studies on the musculoskeletal system using sonoelastography have reported its value in detecting abnormalities. On the other hand, sonoelastography of peripheral nerves has recently begun to be utilized, and it is generally used in the diagnosis of peripheral nerve neuropathies.^[3–6]^ Although there are studies in which the brachial plexus has been assessed, a literature review has not identified a study that evaluates the sonoelastographic assessment of the brachial plexus and the changes in stiffness values based on the head and arm positions.

In this study, we aimed to evaluate the brachial plexus using sonoelastography and to compare stiffness values in the brachial plexus during under 3 different positions: neutral, head rotated, and head rotated with arm hyperabducted.

## 2. Materials and methods

This prospective cohort study was carried out in accordance with the guidelines of the Declaration of Helsinki. After obtaining approval from the Institutional Review Board (B.30.2.ATA.0.01.00/103), registration for clinical trials was made (www.clinicaltrials.gov NCT04959058). The investigation was conducted in the Anesthesiology and Reanimation unit of the Koc University Hospital. Each patient provided his/her consent voluntarily. The Strengthening the Reporting of Observational Studies in Epidemiology statement was used for reporting.

The study included 8 volunteers, American Society of Anesthesiologists I to II, aged 18 and over. Demographic data of the participants, including gender, age, weight, and height, were recorded.

Exclusion criteria for the study included individuals with a history of upper extremity or cervical trauma, muscle weakness, cervical disc herniation, spinal or cranial surgery, neuromuscular disease, a diagnosis of brachial plexopathy, hyperlipidemia, diabetes mellitus, history of radiotherapy in the cervical region, and morbid obesity (body mass index > 35 kg/m^2^). Additionally, an examination of the muscle strength of the upper extremity and cervical muscles was performed before the procedure, and volunteers with muscle weakness were excluded from the study.

High-frequency (13-4 MHz) linear probe *GE Logiq S7* (General Electric Healthcare, Chicago, IL) ultrasound device was used for measurements. The brachial plexus was assessed from the supraclavicular area. To prevent arterial pulse-induced movement from affecting sonoelastography measurements, the probe was advanced cranially until the arterial pulses disappeared after it was seen that all brachial plexus components were united in the supraclavicular region. In addition, measurements were taken from the area where the brachial plexus roots merge and the trunks approach each other.

**Figure 1.**
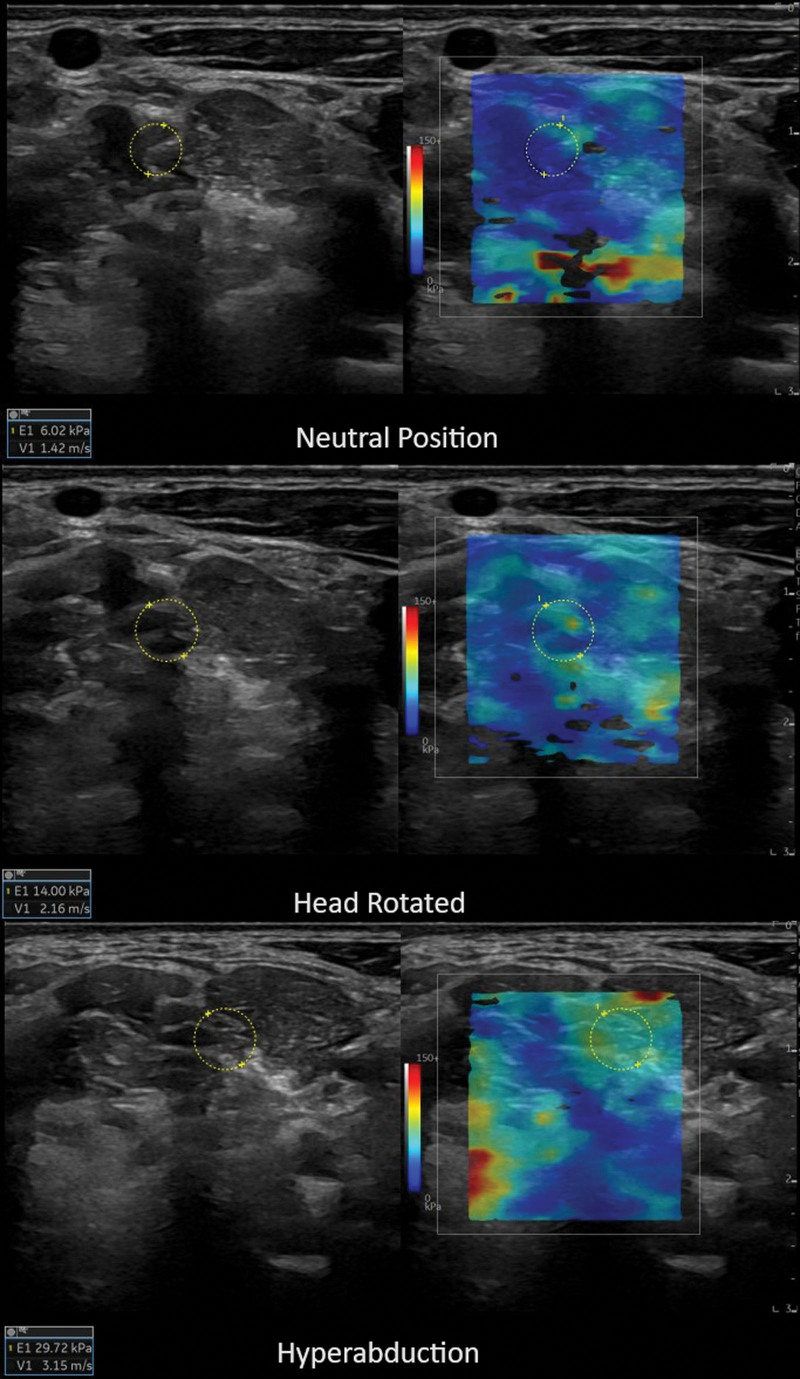
Sonoelastographic images of the brachial plexus in neutral, head turned and hyperabduction states.

Measurements were performed jointly by 2 researchers with at least 5 years of experience in ultrasonography (M.A.K., K.D.). To maintain consistent pressure during each measurement, a holder was used to fix the ultrasound probe to the bed, and to prevent operator-dependent errors, measurements were taken 5 consecutive times. Measurements were taken in 3 different positions. The brachial plexus was visualized while the arm and head were in a neutral position (NP), with the head turned to the opposite side, and with the head turned to the opposite side while the arm was in hyperabduction (HR-HA). The area between the upper and lower limits of the plexus was marked, and stiffness measurements were performed using the shear-wave sonoelastography method. The color box with adequate size was applied and displayed as an area of multiple colors, with blue representing softer tissue and red representing harder tissue. Images were obtained when there were no artifacts and the box fulfilled with color. Quantitative sonoelastographic assessment was performed using a 2 × 2 cm region of interest, with the transducer positioned at the stiffest area. All measurements were recorded and saved in kilopascals (kPa). Five measurements were taken for each brachial plexus, and the mean sonoelastography was recorded (Fig. 1).

### 2.1. Statistical analysis

For an effect size of η^2^ = 0.47, it was calculated with the G power program that 48 plexuses should be examined in 3 different positions in total if there are at least 16 in each position at 80% power and 95% confidence level.

The analyses were performed using the IBM SPSS 20 statistical analysis software. Data were presented as mean, standard deviation, median, minimum, maximum, percentage, and count. The normal distribution of continuous variables was assessed using the Shapiro–Wilk test and the Kolmogorov-Smirnov test. In comparisons between 2 independent groups, the Independent Samples t-test was used when the condition of normal distribution was met, and the Mann–Whitney U test was used when it was not met.

For comparisons involving more than 2 independent groups and continuous variables, the ANOVA test was used when the normal distribution existed, and the Kruskal-Wallis test was used when it did not. post hoc tests after the ANOVA test were performed using the Tukey test when variances were homogeneous and the Tamhane T2 test when variances were not homogeneous. post hoc tests after the Kruskal-Wallis test were performed using the Kruskal-Wallis 1-way ANOVA (k samples) test. The intraclass correlation (ICC) analysis was used for intra-class agreement. The internal consistency among the items of the scales was presented with the Cronbach alpha value. The level of statistical significance was set at *P* < .05.

## 3. Results

Eight adult volunteers (3 males and 5 females) were included in the study. The average age of the participants was calculated as 28 ± 12 years (range, 24–41). The average body mass index of the volunteers was 22.47 ± 7.29 (range, 18.42–29.76). The bilateral brachial plexuses of the volunteers were evaluated (total of 16 brachial plexuses). The stiffness of the brachial plexus was measured in the NP, with the head turned to the right and left, and when the arm was in hyperabduction with the head turned.

In the NP, the average stiffness value of the brachial plexus was measured to be 7.39 kPa. With the head turned, the average stiffness value of the brachial plexus was measured to be 10.28 kPa. When the head was turned to the opposite side and the ipsilateral arm was in hyperabduction, the average stiffness value of the brachial plexus was measured to be 17.24 kPa. A statistically significant increase was found between the stiffness values in the NP and with the head turned to the right (*P* < .001) (Table 1). Similarly, the average stiffness values in positions with the head turned to the left were also found to be statistically significantly higher compared to the NP (*P* < .001) (Table 1). There was a statistically significant difference between the average stiffness values with the head turned to both the right and left sides (*P* < .001) (Table 1).

**Table 1:** Stiffness values of brachial plexus with different head and arm positions.

		Mean	Standard deviation	Minimum	Maximum
Arm	Right	12.11	6.10	4.20	26.00
	Left	11.16	5.08	4.60	25.20
Position	NP	7.39	1.92	4.20	11.20
	HR	10.28	3.19	4.80	17.40
	HR + HA	17.24	5.37	9.60	26.00

HR = while the head is in rotation, HR + HA = With the head in rotation and the arm in hyperabduction, NP = neutral position.

For the right brachial plexus, a strong correlation was found between 2 measurements in the intra-class agreement (Intraclass Correlation Coefficient) of the 5 different stiffness values taken in all positions, and this correlation was found to be statistically significant (ICC = 0.988; *P* < .001; Cronbach Alpha = 0.987) (Table 2).

**Table 2:** Intraclass correlation between the measurements of right and left brachial plexus.

Brachial plexus		Cronbach alpha	Intraclass correlation	95% confidence interval	
				Lower bound	Upper bound
Right	Average Measures	0.987	0.988	0.978	0.994
Left	Average Measures	0.989	0.989	0.980	0.995
Right&Left	Average Measures	0.988	0.988	0.982	0.993

For the left brachial plexus, a strong correlation was found between 2 measurements in the intra-class agreement (Intraclass Correlation Coefficient) of the 5 different stiffness values taken in all positions, and this correlation was found to be statistically significant (ICC = 0.989; *P* < .001; Cronbach Alpha = 0.989) (Table 2).

For both right and left brachial plexuses, a strong correlation was found between 2 measurements in the intra-class agreement (Intraclass Correlation Coefficient) of the 5 different stiffness values taken in all positions, and this correlation was found to be statistically significant (ICC = 0.988; *P* < .001; Cronbach Alpha = 0.988) (Table 2).

## 4. Discussion

In this study, we compared the changes in the stiffness values of the brachial plexus as measured by sonoelastography under various head positions and when hyperabduction of the arm was added to these positions. According to the study findings, the brachial plexus is stretched in all but the NP of the head. The strongest manifestation of this effect occurred when the head was turned to the other side while the arm was hyperabducted.

Brachial plexus injuries, like other neuropathies, usually occur because of stretching, ischemia, and increased pressure.^[1]^ It is hypothesized that prolonged stretching of a strained nerve impairs its microvascular circulation, thereby jeopardizing the nourishment of the nerve fibers. Animal studies have shown that when nerves are stretched by 15% or more of their resting lengths, intraneural microcirculation may be compromised.^[7]^ Nerve cells are sensitive to ischemia, so events that interfere with intraneural blood flow can cause nerve dysfunction.^[7]^ Although axons, a component of nerve cells, are resistant to ischemia, prolonged stretching can lead to damage in them as well. When mechanical trauma due to stretching is added to ischemic changes, this hypoxic condition can also cause damage in axons. Prolonged stretching can lead to extravasation of proteins and intrafascicular edema once the nerve relaxes and blood flow is restored, due to damage inflicted on the blood vessel walls.^[7]^

During surgery, one of the nerves most frequently damaged is the brachial plexus.^[1]^ The brachial plexus is vulnerable due to its superficial location, proximity to bony structures, relatively long course, and its path through the gap between the clavicle and the first rib. Under general anesthesia, stretching injuries occur most frequently, with compression injuries occurring less commonly.^[8]^ Therefore, in anesthetized patients, care should be taken to properly position the arm and shoulder without causing trauma.

The American Society of Anesthesiologists recommends limiting arm abduction to 90 degrees when the forearm is in a NP for patients lying on their backs.^[9]^ However, it is known that even 90 degrees of abduction can cause damage if the procedure is prolonged.^[8,10]^ In our study, we assessed the stiffness in the brachial plexus immediately after the patients perform hyperabduction beyond 90 degrees. The stiffness values measured from the nerve in hyperabduction were statistically significantly higher compared to the NP although not for a long time.

Elasticity is defined as the ability to return to its original size and shape after being subjected to a deforming force or stress. Sonoelastography is an imaging method that can evaluate the elasticity of tissues with similar echo patterns, enhancing the diagnostic performance of B-mode ultrasonography. Recently, this method has been used to differentiate abnormalities involving malignancies, fibrosis, and inflammatory changes in different organs.^[6,11,12]^ Our measurements of stiffness values from all patients’ brachial plexuses using sonoelastography suggest that this technique may predict potential future damage for healthy young volunteers as well.

There are 2 main techniques for sonoelastography: compression elastography (CE) and shear wave elastography (SWE). The main limitations of CE are the lack of standardized and repeatable measurements and threshold values, operator dependence, and the need for a reference tissue for strain ratio calculation.^[11]^ SWE is a less operator-dependent method and has been developed to overcome these limitations.^[13,14]^ SWE allows for the visualization and documentation of absolute stiffness, in kPa or meters per second, without any manipulation.^[13]^ The main strength of our study is that, we used SWE and employed a tablet holder to minimize operator dependence, which contributed to our intra-class correlation.

In a study to determine the reliability of SWE and strain elastography measurements for the normal brachial plexus and sonoelastographic findings, the mean normal stiffness value of the brachial plexus was measured as 17.03, 15.03, and 13.83 kPa. They performed SWE while the participant was in the supine position; the head was tilted and turned slightly to the other side while the arms were in a neutral anatomic position.^[6]^ The main contribution of our study is the measurement of the mean stiffness value of the brachial plexus in the NP (7.39 kPa) A study evaluating different musculoskeletal disorders showed the changes in nerve stiffness with movement. They emphasized the importance of the effects of extremity position, age, and nerve tension on the nervous system.^[15]^ Our study also supports these results and can inspire further studies/technologies for the aim of monitorization of the nerves especially under prolonged anesthesia.

In a study in which the brachial plexus was evaluated by sonoelastography, the C5, C6, and C7 nerve roots were visualized with a longitudinal view.^[16]^ For each root individual sonoelastography measurements were taken centrally, in the area where the nerve roots immediately separate from the medulla spinalis. Their stiffness values were higher than our results by in NP. The reason for that could be the location where the more abundant and dense connective tissue closer to the vertebral column. Our hypothesis was that the brachial plexus was affected by the movements of the head and arm, especially in the supraclavicular region. Similar research was done using passive movement on the sciatic nerve in the lower extremity.^[17]^ It has been demonstrated that as an extremity moves, the stiffness values obtained from sonoelastography alter statistically significantly.

The limitations of our study include the small number of patients, potential population bias and being single-centered. Furthermore, it is considered that pulsations related to the subclavian artery might affect the diagnostic performance of SWE. Additionally, only healthy volunteers were enrolled. Although the ability to measure in different positions is one of the strengths of the study, the fact that the measurements were not made during the surgery itself may be a possible population bias. In addition, the fact that no evaluation was made under anesthesia, especially in the case of administration of muscle relaxants or after a certain period, limitations of the study.

## 5. Conclusion

Brachial plexus injuries are one of the significant surgical complications resulting from patient positioning. Sonoelastography has demonstrated an increase in the stiffness values of the brachial plexus when the head is rotated, and during hyperabduction of the arm while the head is rotated, compared to its NP. Similar studies conducted in larger groups are promising, especially for the development of a potential cutoff value that may, in the future, prove contributory in minimizing the risk of injury.

## Author contributions

**Conceptualization:** Muhammet Ahmet Karakaya, Aysenur Dostbil.

**Data curation:** Muhammet Ahmet Karakaya, Kamil Darcin, Yilmaz Yenigun, Kamber Kasali.

**Formal analysis:** Muhammet Ahmet Karakaya, Kamber Kasali.

**Investigation:** Muhammet Ahmet Karakaya.

**Methodology:** Muhammet Ahmet Karakaya, Ilker Ince, Aysenur Dostbil.

**Supervision:** Muhammet Ahmet Karakaya, Kamil Darcin, Ilker Ince, Aysenur Dostbil.

**Validation:** Muhammet Ahmet Karakaya.

**Visualization:** Muhammet Ahmet Karakaya, Yilmaz Yenigun.

**Writing – original draft:** Muhammet Ahmet Karakaya.

**Writing – review & editing:** Muhammet Ahmet Karakaya.

## References

[1] Winfree CJ Kline DG. Intraoperative positioning nerve injuries. Surg Neurol. 2005;63:5–18; discussion 18. 15639509 10.1016/j.surneu.2004.03.024

[2] Wee TC Simon NG. Ultrasound elastography for the evaluation of peripheral nerves: a systematic review. Muscle Nerve. 2019;60:501–12. 31269240 10.1002/mus.26624

[3] Kantarci F Ustabasioglu FE Delil S. Median nerve stiffness measurement by shear wave elastography: a potential sonographic method in the diagnosis of carpal tunnel syndrome. Eur Radiol. 2014;24:434–40. 24220753 10.1007/s00330-013-3023-7

[4] Wells PN Liang HD. Medical ultrasound: imaging of soft tissue strain and elasticity. J R Soc Interface. 2011;8:1521–49. 21680780 10.1098/rsif.2011.0054 PMC3177611

[5] Botanlioglu H Kantarci F Kaynak G. Shear wave elastography properties of vastus lateralis and vastus medialis obliquus muscles in normal subjects and female patients with patellofemoral pain syndrome. Skeletal Radiol. 2013;42:659–66. 22996306 10.1007/s00256-012-1520-4

[6] Aslan A Aktan A Aslan M. Shear wave and strain elastographic features of the brachial plexus in healthy adults: reliability of the findings: a pilot study. J Ultrasound Med. 2018;37:2353–62. 29480537 10.1002/jum.14584

[7] Lundborg G Rydevik B. Effects of stretching the tibial nerve of the rabbit. J Bone Joint Surg Br. 1973;55:390–401. 4707307

[8] Zhang J Moore AE Stringer MD. Iatrogenic upper limb nerve injuries: a systematic review. ANZ J Surg. 2011;81:227–36. 21418465 10.1111/j.1445-2197.2010.05597.x

[9] ASA Task Force on prevention of perioperative peripheral neuropathies. Practice advisory for the prevention of perioperative peripheral neuropathies. Anesthesiology. 2000;92:1168–82. 10754638 10.1097/00000542-200004000-00036

[10] Hida A Arai T Nakanishi K. Bilateral brachial plexus injury after liver transplantation. J Anesth. 2008;22:308–11. 18685942 10.1007/s00540-008-0636-0

[11] İnal M Tan S Yumusak EM. Evaluation of the optic nerve using strain and shear wave elastography in patients with multiple sclerosis and healthy subjects. Med Ultrason. 2017;19:39–44. 28180195 10.11152/mu-939

[12] Abdellah MMH Bamidele JO Debbage P. Future of musculoskeletal ultrasound. Curr Radiol Rep. 2015;3:21.

[13] Yang YP Xu XH Guo LH. Qualitative and quantitative analysis with a novel shear wave speed imaging for differential diagnosis of breast lesions. Sci Rep. 2017;7:40964. 28102328 10.1038/srep40964 PMC5244419

[14] Alfuraih AM O’Connor P Hensor E. The effect of unit, depth, and probe load on the reliability of muscle shear wave elastography: variables affecting reliability of SWE. J Clin Ultrasound. 2018;46:108–15. 28990683 10.1002/jcu.22534

[15] Greening J Dilley A. Posture-induced changes in peripheral nerve stiffness measured by ultrasound shear-wave elastography. Muscle Nerve. 2017;55:213–22. 27396239 10.1002/mus.25245

[16] Bedewi MA Nissman D Aldossary NM. Shear wave elastography of the brachial plexus roots at the interscalene groove. Neurol Res. 2018;40:805–10. 29873619 10.1080/01616412.2018.1480922

[17] Andrade RJ Nordez A Hug F. Non-invasive assessment of sciatic nerve stiffness during human ankle motion using ultrasound shear wave elastography. J Biomech. 2016;49:326–31. 26725218 10.1016/j.jbiomech.2015.12.017

